# Diseases of the Urinary Apparatus

**Published:** 1892-10

**Authors:** 


DISEASES OF THE URINARY APPARATUS. Phlegmasic Affections. By W. S. Gouley, M. D., Surgeon to Bellevue Hospital. Pp. 432. New York: D. Appleton & Co. 1892.

The matter of this book was published last year in the *N. Y. Medical Journal*, and has been revised and published in this form as a contribution to the pathology and treatment, principally of phlegmasic affections of the urinary apparatus. It gives a good resumé of the subject. B.

